# Association between urinary free light chains and progression to end stage renal disease in chronic kidney disease

**DOI:** 10.1371/journal.pone.0197043

**Published:** 2018-05-09

**Authors:** Anthony Fenton, Mark D. Jesky, Rachel Webster, Stephanie J. Stringer, Punit Yadav, Iain Chapple, Indranil Dasgupta, Stephen J. Harding, Charles J. Ferro, Paul Cockwell

**Affiliations:** 1 Department of Renal Medicine, University Hospitals Birmingham NHS Foundation Trust, Birmingham, United Kingdom; 2 Institute of Inflammation and Ageing, University of Birmingham, Birmingham, United Kingdom; 3 Department of Biochemistry, University Hospitals Birmingham NHS Foundation Trust, Birmingham, United Kingdom; 4 Periodontal Research Group, Institute of Clinical Sciences, University of Birmingham and Birmingham Community Healthcare Foundation Trust, Birmingham, United Kingdom; 5 Department of Renal Medicine, Heart of England NHS Foundation Trust, Birmingham, United Kingdom; 6 The Binding Site, Edgbaston, Birmingham, United Kingdom; University of Colorado Denver School of Medicine, UNITED STATES

## Abstract

**Background:**

Patients with chronic kidney disease (CKD) are at an increased risk of developing end-stage renal disease (ESRD). We assessed for the first time whether urinary free light chains (FLC) are independently associated with risk of ESRD in patients with CKD, and whether they offer incremental value in risk stratification.

**Materials and methods:**

We measured urinary FLCs in 556 patients with CKD from a prospective cohort study. The association between urinary kappa/creatinine (KCR) and lambda/creatinine (LCR) ratios and development of ESRD was assessed by competing-risks regression (to account for the competing risk of death). The change in C-statistic and integrated discrimination improvement were used to assess the incremental value of adding KCR or LCR to the Kidney Failure Risk Equation (KFRE).

**Results:**

136 participants developed ESRD during a median follow-up time of 51 months. Significant associations between KCR and LCR and risk of ESRD became non-significant after adjustment for estimated glomerular filtration rate (eGFR) and albumin/creatinine ratio (ACR), although having a KCR or LCR >75^th^ centile remained independently associated with risk of ESRD. Neither KCR nor LCR as continuous or categorical variables provided incremental value when added to the KFRE for estimating risk of ESRD at two years.

**Conclusions:**

Urinary FLCs have an association with progression to ESRD in patients with CKD which appears to be explained to a degree by their correlation with eGFR and ACR. Levels above the 75^th^ centile do have an independent association with ESRD, but do not improve upon a current model for risk stratification.

## Introduction

Patients with chronic kidney disease (CKD) are at an increased risk of adverse health outcomes including progression to end-stage renal disease (ESRD) and early mortality [1–6]. Risk prediction models can be used to estimate an individual’s risk of ESRD, incorporating prognostic factors such as age, gender, glomerular filtration rate (GFR), and level of albuminuria [7–9]. There is significant interest in identifying further prognostic factors, including urinary proteins in addition to albumin, with a view to enhancing prognostic models and identifying potential targets for novel interventions.

During the synthesis of intact immunoglobulins, light chains are produced in excess of heavy chains, resulting in the release into the circulation of approximately 500 mg per day of unbound free light chains (FLC) [10]. As monomeric FLCs (usually kappa) weigh ~25 kDa, and dimeric FLCs (usually lambda) weigh ~50 kDa, they are filtered at the glomerulus and then reabsorbed and metabolised in the proximal tubule [11]. Although small amounts of FLC may be present from mucosal secretion from the urinary tract, the presence of significant FLC in the urine implies either concentrations in the proximal tubule greater than can be reabsorbed, as may occur with excess monoclonal FLC production in plasma cell dyscrasias, or renal disease with glomerular hyperfiltration and/or tubular dysfunction [12].

Previous work has demonstrated that urinary FLC concentration increases as estimated GFR (eGFR) decreases, and urinary FLC may be more sensitive than albuminuria as a marker of early CKD [13]. In patients with type 2 diabetes mellitus (DM), raised urinary concentrations of FLC are detectable prior to the development of increased albuminuria [14, 15]. Urinary FLC levels have also been shown to correlate with disease activity in IgA nephropathy and lupus nephritis [16, 17].

Our objectives were to assess, for the first time, whether urinary FLC are independently associated with risk of ESRD in patients with CKD, and whether measuring urinary FLC improves upon an established model for risk stratification.

## Materials and methods

We used data and samples from the Renal Impairment in Secondary Care (RIISC) Study (ClinicalTrials.gov: NCT01722383). The RIISC study is a prospective cohort study of patients with CKD and the detailed methodology has been published previously [18].

In brief, patients in nephrology clinics at two renal centres were invited to participate if they had high-risk CKD, defined as an eGFR < 30 mL/min/1.73m^2^, or an eGFR 30–59 mL/min/1.73m^2^ with a decline of ≥ 5 mL/min/1.73m^2^ over a year or ≥ 10 mL/min/1.73m^2^ over 5 years or an ACR ≥ 70 mg/mmol on three occasions. Patients were excluded if they had received immunosuppression for immune-mediated renal disease or if they had started renal replacement therapy (RRT, i.e. dialysis or kidney transplant). For this analysis, we also excluded patients with a monoclonal gammopathy i.e. monoclonal gammopathy of undetermined significance (MGUS), including patients with an abnormal serum FLC isotype and a serum kappa/lambda (κ/λ) FLC ratio outside of the renal reference range (0.37–3.1) [13, 19], or a known diagnosis of myeloma, AL amyloidosis, or other monoclonal gammopathy of renal significance. Participants were recruited between October 2010 and December 2015 and followed up until they started RRT or died. The primary outcome of interest was progression to ESRD, which was defined as the initiation of RRT and was captured using both centres’ local databases of patients who have started dialysis or received a kidney transplant. Outcomes were captured up to 31 January 2017 and patients who had not reached a study end-point were censored on this date. Ethical approval was granted by the South Birmingham Research Ethics Committee (reference: 10/H1207/6). All patients provided written consent, and the study was conducted in accordance with the Declaration of Helsinki.

### Laboratory analyses

Serum and urine were processed immediately after collection according to pre-defined standard operating procedures and stored at -80°C until analysis.

Urinary FLCs were measured by turbidimetry on a Roche Modular P analyser using the Freelite^™^ immunoassay (The Binding Site Group Ltd, Birmingham, UK). To correct for variations in urine concentration, urinary FLCs were divided by urine creatinine concentration to give urinary FLC/creatinine (FLC/Cr) ratios in mg/mmol, i.e. a kappa/creatinine ratio (KCR) and a lambda/creatinine ratio (LCR). Serum creatinine measurements were performed on a Roche Modular analyser using a rate-blanked and compensated Jaffe reaction, and eGFR was calculated using the Chronic Kidney Disease Epidemiology Collaboration (CKD-EPI) equation [20]. Serum kappa (κ) and lambda (λ) FLC concentrations were measured by nephelometry on a Dade-Behring BN^™^ II System (Siemens AG, Erlangen, Germany) using the Freelite^™^ assay. Urine ACR was measured using a Roche Hitachi 702 analyser. Other biochemistry testing was performed by the local clinical laboratories in accordance with the current standard of care.

### Blood pressure

Blood pressure (BP) was measured using the BpTRU automated device (BpTRU Medical Devices, Coquitlam, BC, Canada) which obtains six BP readings after a five-minute rest period. The systolic (SBP) and diastolic BP (DBP) are derived from the mean of the second to sixth readings, and this method has been reported to be comparable to the mean daytime BP from 24-hour ambulatory BP monitoring [21]. Mean arterial pressure (MAP) was calculated as ((2 × *DBP*) + *SBP*) ÷ 3.

### Statistical analyses

Statistical analyses were performed using SPSS Statistics 24 (Armonk, NY: IBM Corp, 2016) and Stata 15 (College Station, TX: StataCorp, 2017). Baseline characteristics are presented as a frequency and percentage for categorical variables, and a median and interquartile range (IQR) for continuous variables.

Urinary FLC/Cr ratios were log-transformed prior to parametric testing. Their associations with other continuous baseline variables were examined by scatter plots and Pearson’s correlation coefficients. Correlation coefficients of 0.2, 0.5, and 0.8 were considered weak, moderate, and strong, respectively [22]. To assess their association with categorical variables, between-group differences were tested for using the Mann-Whitney U (two groups) and Kruskal-Wallis (three or more groups) tests. Multiple linear regression was used to further assess the influence of serum FLC on urinary FLC/Cr ratios after adjustment for other significant determinants of urinary FLC/Cr ratios (eGFR, ACR, DM, and renal diagnosis).

The associations between urinary FLC/Cr and progression to ESRD were assessed in univariable and multivariable regression models. As 75 (13.5%) participants died without ESRD, we used competing-risks regression based on the proportional subhazards model of Fine and Gray [23], which allowed us to handle mortality as a competing event (simply censoring at death in standard Cox regression models would have made the incorrect assumption that the censored deceased participants remained at risk of developing ESRD). In the multivariable models, we adjusted for confounding factors known to be associated with CKD progression: eGFR (model 1); ACR (model 2); eGFR and ACR (model 3); age, gender, ethnicity, renal diagnosis, MAP, eGFR, and ACR (model 4). Subhazard ratios (SHR) are presented with 95% confidence intervals (CI), which for continuous variables represented the risk of ESRD associated with an increase of one standard deviation (SD). Urinary FLC/Cr ratios were analysed as both full-range continuous variables and as categorical variables (comparing above versus below the 75^th^ centiles). Competing-risks models were also built to include restricted cubic splines of urinary FLC/Cr ratios to assess for non-linear associations between urinary FLC/Cr ratios and risk of ESRD. Kaplan-Meier curves were plotted to show the cumulative incidence of ESRD by quartiles of urinary FLC/Cr.

To examine whether urinary FLC/Cr provide any incremental value in risk stratification, we chose the four-variable ‘Kidney Failure Risk Equation’ (KFRE) [9] as the baseline model for comparison, which estimates an individuals’ risk of ESRD at two and five years. Binary logistic regression models were fitted for the outcome of ESRD at two years. The baseline model contained only the KFRE-calculated two-year risk of ESRD, calculated as:

$${1–0.9832}^{e^{\left(-0.2201\;\times \;(age/10–7.036)\;+\;0.2467\;\times \;(male\;–\;0.5642)–\;0.5567\;\times \;(eGFR/5–7.222)\;+\;0.4510\;\times \;(logACR\;–\;5.137)\right)}}$$

(the four-variable, non-North America, two-year risk equation from eAppendix 2 of [24]; ACR was converted to mg/g before being entered into the model by dividing by 0.113).

KCR and LCR (separately) were added as continuous and categorical predictors to the baseline KFRE model and the models compared. Overall model performance was estimated by *R*^2^ (Nagelkerke, a version of the Cox & Snell *R*^2^ adjusted to cover the full range from 0 to 1 [25]), discrimination was assessed by the C-statistic, and calibration by the Hosmer-Lemeshow goodness-of-fit test. The incremental value of adding KCR or LCR to the baseline model was assessed by the change in C-statistic and by the reclassification measure Integrated Discrimination Index (IDI). The IDI is a measure of the extent to which adding a new marker to a model correctly revises upward the predicted risk of individuals who experience an event and correctly revises downward the predicted risk of individuals who do not experience an event [26, 27].

## Results

Urinary FLCs were measured in 636 participants of the RIISC study. We excluded 41 patients with a monoclonal gammopathy (21 with a serum κ/λ FLC ratio outside the renal reference range, 15 with MGUS, and 5 with multiple myeloma), and also excluded 39 patients with urinary FLC or creatinine results above or below the limits of detection so that accurate FLC/Cr ratios could be calculated. Therefore, 556 participants were included for analysis with a median follow-up time of 51 (IQR 46–60) months.

### Baseline characteristics

The baseline characteristics of the study population are shown in Table 1. The cohort had a median age of 64 (IQR 51–76) years, were 63% male, and 68% were of White ethnicity. The most common aetiologies of CKD were ischaemic/hypertensive nephropathy (28.9%), glomerulonephritis (14.3%), and diabetic kidney disease (12.9%). Median eGFR was 25 (IQR 19–34) mL/min/1.73m^2^ and median ACR was 28.1 (IQR 5.8–103.2) mg/mmol. Median urinary KCR was 14.6 (IQR 7.1–27.7) mg/mmol and median LCR was 2.1 (IQR 1.0–5.1) mg/mmol.

**Table 1:** Baseline characteristics of the study population (*N* = 556).

Characteristic	Median (IQR) or *N* (%)	Completeness of data (%)
**Age (years)**	64 (51–76)	100
**Male gender**	351 (63.1)	100
**Ethnicity**		100
White	380 (68.3)	
South Asian	117 (21.0)	
Black	56 (10.1)	
Other	3 (0.5)	
**Co-morbidities**		100
Diabetes mellitus	196 (35.3)	
Ischaemic heart disease	120 (21.6)	
Cerebrovascular disease	53 (9.5)	
Peripheral vascular disease	53 (9.5)	
COPD	57 (10.3)	
Malignancy	71 (12.8)	
**Renal diagnosis**		90
Ischaemic/hypertensive	145 (28.9)	
Glomerulonephritis	72 (14.3)	
Diabetic kidney disease	65 (12.9)	
Polycystic kidney disease	29 (5.8)	
Interstitial nephropathy	29 (5.8)	
Reflux nephropathy	12 (2.4)	
Other/uncertain	150 (29.9)	
**Estimated GFR (mL/min/1.73m**^**2**^**)**	25 (19–34)	98
**Urinary ACR (mg/mmol)**	28.1 (5.7–103.2)	92
**Systolic blood pressure (mmHg)**	128 (116–144)	99
**Diastolic blood pressure (mmHg)**	76 (68–85)	99
**Serum κ FLC (mg/L)**	44.9 (29.5–67.0)	99
**Serum λ FLC (mg/L)**	32.5 (23.4–47.0)	99
**Urinary KCR (mg/mmol)**	14.6 (7.1–27.7)	100
**Urinary LCR (mg/mmol)**	2.1 (1.0–5.1)	100

Continuous variables are expressed as a median and interquartile range, and categorical variables are expressed as a frequency and percentage.

Abbreviations: ACR = albumin/creatinine ratio; COPD = chronic obstructive pulmonary disease; FLC = free light chains; GFR = glomerular filtration rate; IQR = interquartile range; KCR = kappa/creatinine ratio; LCR = lambda/creatinine ratio.

### Urinary FLC/Cr associations

The associations between urinary KCR and LCR with other baseline variables are shown in Table 2. For comparison, the associations with ACR are also given. Urinary FLC/Cr ratios had moderate positive correlations with serum FLC (sFLC), weak-to-moderate positive correlations with ACR, and weak negative correlations with eGFR. The urinary FLC/Cr ratios, unlike ACR, increased significantly with worsening CKD stage.

**Table 2:** Associations between urinary free light chain/creatinine ratios and albumin/creatinine ratio with other baseline variables.

Variable	Urinary kappa/creatinine ratio		Urinary lambda/creatinine ratio		Urinary albumin/creatinine ratio	
	Median (IQR) or *r*	*P*	Median (IQR) or *r*	*P*	Median (IQR) or *r*	*P*
**Age**	0.109	0.010	0.012	0.77	-0.321	<0.0001
**Gender**		0.028		0.007		0.038
Male	16.2 (7.4–29.3)		2.3 (1.1–5.2)		32.5 (7.3–111.3)	
Female	12.3 (6.3–26.5)		1.9 (0.7–4.3)		20.0 (4.4–83.2)	
**Ethnicity**		0.007		0.001		<0.0001
White	13.1 (7.1–24.5)		1.9 (1.0–4.3)		16.9 (4.2–76.4)	
South Asian	20.0 (8.4–37.2)		3.4 (1.5–7.9)		78.2 (22.8–156.6)	
Black	12.9 (4.0–28.9)		1.8 (0.6–5.2)		39.0 (9.5–88.7)	
Other	12.7 (8.3–39.9)		2.0 (1.9–7.4)		237.3 (187.1–302.4)	
**Co-morbidities**						
**Diabetes Mellitus**		<0.0001		0.001		0.28
Yes	18.7 (8.1–34.9)		2.9 (1.2–6.4)		23.8 (4.2–86.2)	
No	12.6 (6.6–24.3)		1.8 (0.9–4.3)		29.3 (6.9–108.7)	
**Cardiovascular disease**		0.37		0.89		0.048
Yes	16.3 (7.1–30.3)		2.2 (1.0–5.2)		22.6 (3.7–82.6)	
No	14.3 (7.1–27.3)		2.0 (1.0–5.0)		29.1 (6.9–117.0)	
**Malignancy**		0.46		0.35		0.015
Yes	16.0 (7.5–25.9)		1.8 (1.1–3.6)		12.0 (2.9–83.0)	
No	14.3 (7.0–27.8)		2.2 (1.0–5.1)		29.9 (6.5–106.6)	
**Renal diagnosis**		<0.0001		<0.0001		<0.0001
Ischaemic/hypertensive	13.6 (7.3–27.7)		2.1 (1.0–4.7)		12.4 (2.6–56.0)	
Glomerulonephritis	8.2 (4.9–17.2)		1.4 (0.8–2.7)		70.5 (30.4–159.3)	
Diabetic kidney disease	20.9 (10.4–51.8)		4.7 (1.6–8.1)		64.9 (23.0–237.3)	
Polycystic kidney disease	12.0 (5.5–19.0)		1.5 (0.5–3.1)		10.2 (6.1–18.7)	
Interstitial nephropathy	15.0 (8.1–27.3)		3.1 (1.1–5.0)		10.4 (3.6–35.0)	
Reflux nephropathy	8.2 (3.7–22.8)		1.8 (0.6–3.9)		87.8 (29.3–141.0)	
Other/uncertain	16.1 (7.4–31.0)		2.3 (1.0–5.7)		32.9 (6.9–113.5)	
**Estimated GFR**	-0.387	<0.0001	-0.340	<0.0001	0.100	0.027
**CKD Stage**		<0.0001		<0.0001		0.22
G3a	8.6 (4.4–14.0)		1.3 (0.4–2.9)		23.0 (5.1–136.8)	
G3b	12.6 (6.3–22.5)		1.8 (0.8–3.7)		27.9 (7.0–119.8)	
G4	15.2 (7.5–27.8)		2.1 (1.0–5.1)		19.9 (4.4–78.0)	
G5	29.5 (16.3–50.9)		5.3 (2.5–11.0)		33.2 (10.1–117.1)	
**Urinary ACR**	0.400	<0.0001	0.516	<0.0001	N/A	
**Systolic blood pressure**	0.184	<0.0001	0.179	<0.0001	0.227	<0.0001
**Diastolic blood pressure**	0.079	0.06	0.111	0.009	0.231	<0.0001
**Serum κ FLC**	0.513	<0.0001	0.479	<0.0001	0.175	<0.0001
**Serum λ FLC**	0.494	<0.0001	0.563	<0.0001	0.221	<0.0001
**Urinary KCR**	N/A		0.925	<0.0001	0.400	<0.0001
**Urinary LCR**	0.925	<0.0001	N/A		0.516	<0.0001

Associations with continuous variables are expressed as Pearson’s *r* (after log transformation of both variables) and its corresponding *P*. For categorical variables, median and interquartile ranges are shown with between-group differences assessed using the Mann-Whitney U or Kruskal-Wallis tests.

Abbreviations: ACR = albumin/creatinine ratio; CKD = chronic kidney disease; FLC = free light chains; GFR = glomerular filtration rate; IQR = interquartile range; KCR = kappa/creatinine ratio; LCR = lambda/creatinine ratio.

Urinary FLC/Cr ratios were significantly higher in males, those of South Asian ethnicity, and those with diabetic kidney disease, and significantly lower in those with glomerulonephritis and polycystic kidney disease. However, these associations were no longer significant after adjustment for ACR and eGFR. Patients with DM as a comorbidity had significantly higher urinary FLC/Cr ratios, even after adjustment for eGFR, ACR, and serum FLC.

**Fig 1:**
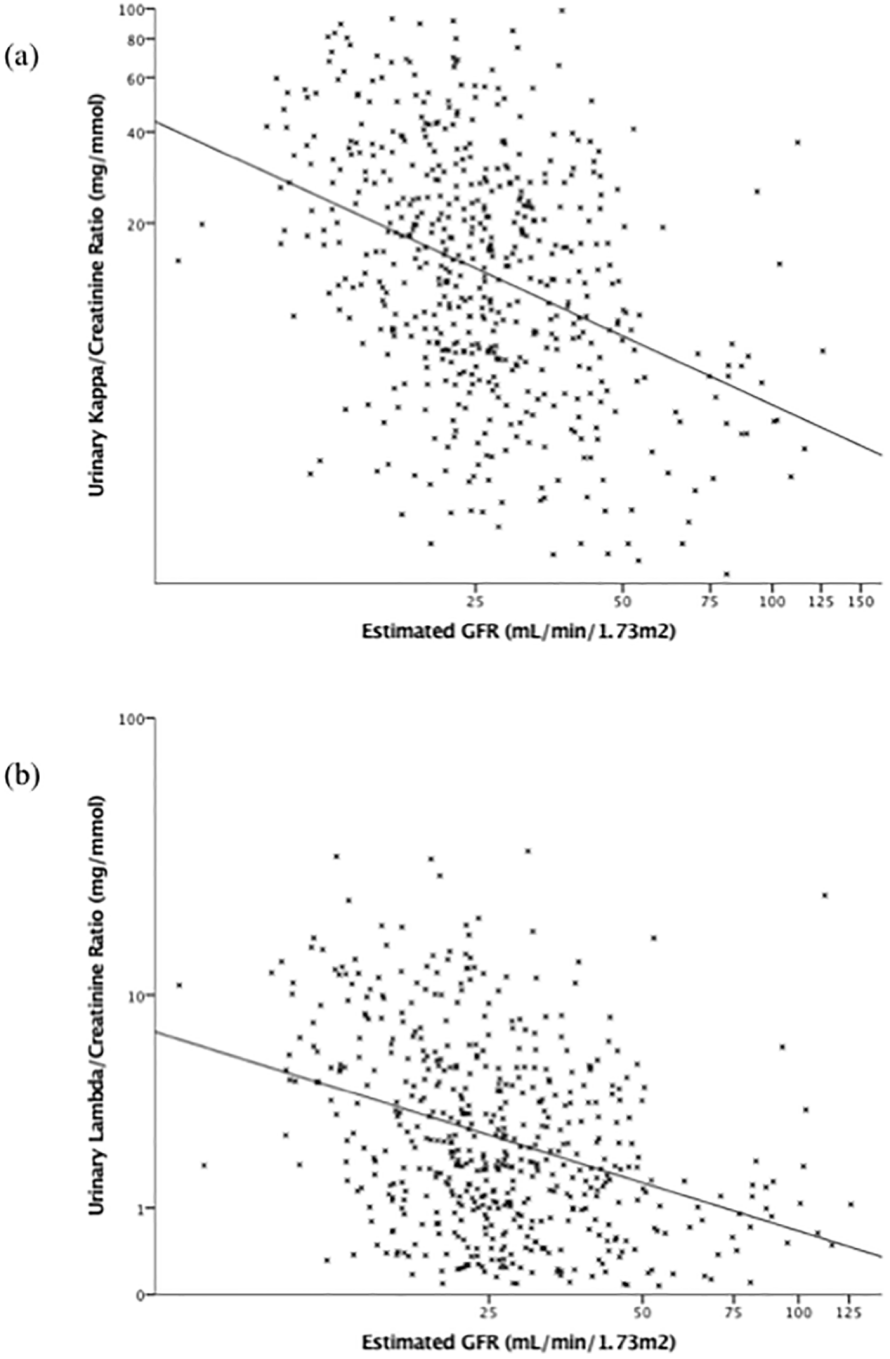
Scatter plots of urinary kappa/creatinine ratio (a) and lambda/creatinine ratio (b) by estimated glomerular filtration rate. Scales are logarithmic. Abbreviations: GFR = glomerular filtration rate.

**Fig 2:**
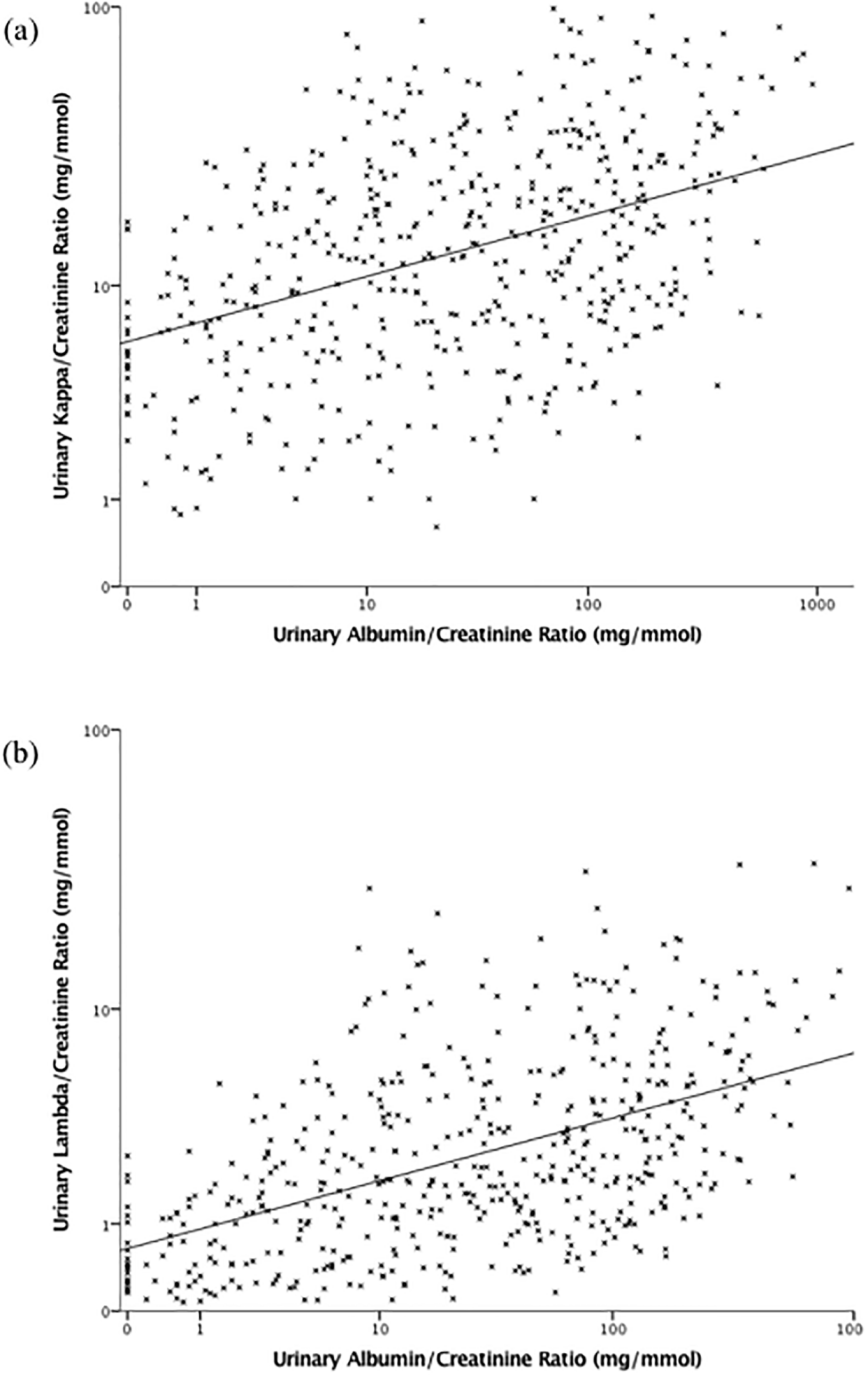
Scatter plots of urinary kappa/creatinine ratio (a) and lambda/creatinine ratio (b) by urinary albumin/creatinine ratio. Scales are logarithmic.

**Fig 3:**
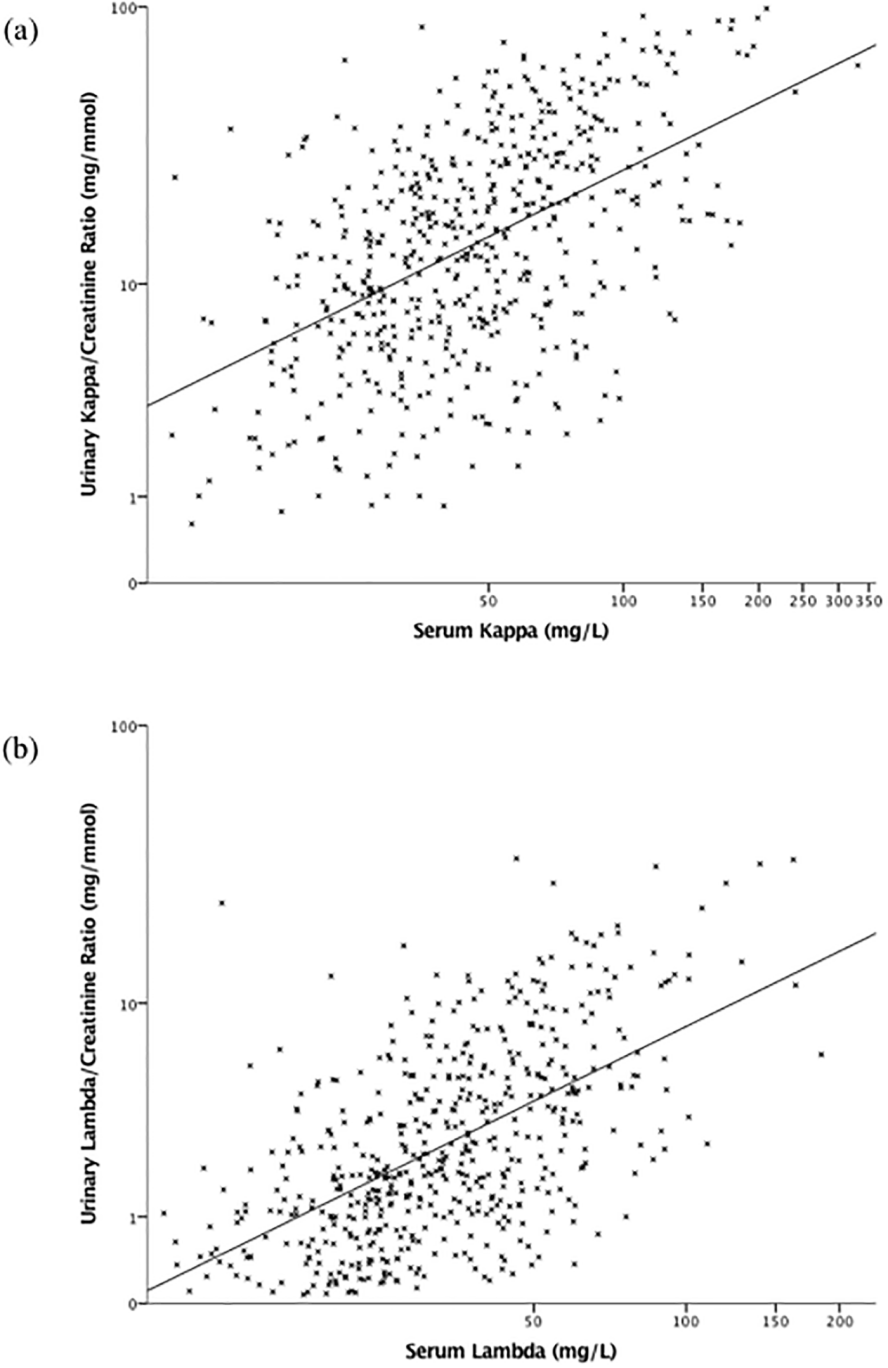
Scatter plots of urinary kappa/creatinine ratio by serum kappa (a) and urinary lambda/creatinine ratio by serum lambda (b). Scales are logarithmic.

Scatter plots of the relationship between urinary FLC/Cr ratios with eGFR, ACR, and serum FLC, are shown in Figs 1, 2 and 3, respectively. Urinary FLC/Cr ratios were most strongly correlated with serum FLC; after adjustment for eGFR, ACR, DM, and renal diagnosis, a 10% higher serum kappa was associated with a 4.8% (3.4–6.2%) higher urinary KCR, and a 10% higher serum lambda was associated with a 7.5% (5.8–9.3%) higher urinary LCR.

### Progression to ESRD

**Fig 4:**
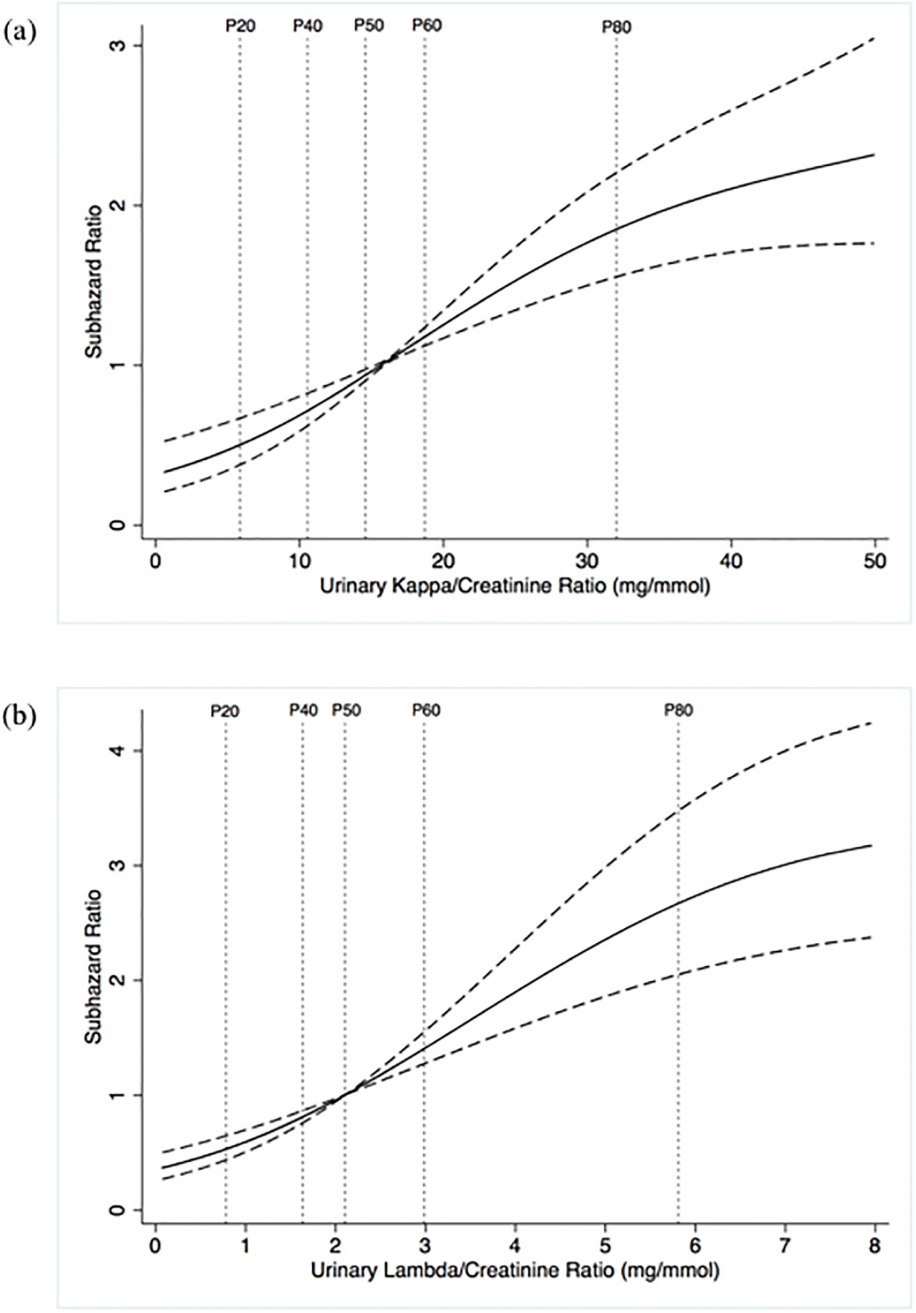
Restricted cubic splines of the subhazard ratio (solid line, with 95% confidence intervals shown as interrupted lines) of end stage renal disease by urinary kappa/creatinine ratio (a) and urinary lambda/creatinine ratio (b), adjusted for the competing risk of death. Percentiles are shown as vertical dotted lines. Abbreviations: P = percentile.

**Fig 5:**
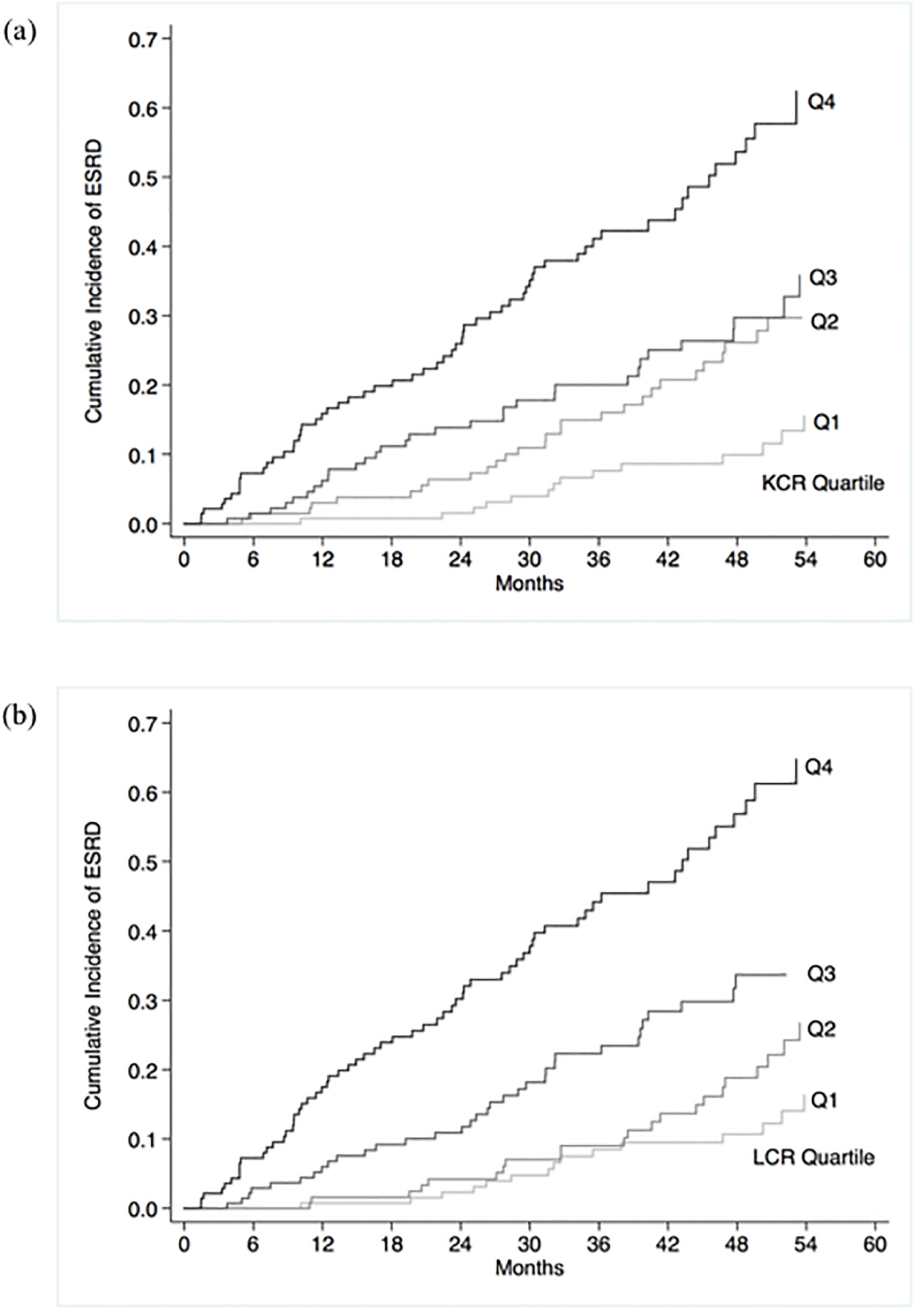
Cumulative incidence of end stage renal disease by quartiles of urinary kappa/creatinine ratio (a) and lambda/creatinine ratio (b). Abbreviations: ESRD = end stage renal disease; KCR = kappa/creatinine ratio; LCR = lambda/creatinine ratio; Q = quartile.

During follow-up, 136 (24.5%) participants progressed to ESRD. A higher KCR and LCR were both associated with a significantly increased risk of ESRD. The association between urinary FLC/Cr ratios and risk of ESRD approximated a linear relationship, although there was a smaller increase in risk per unit increase in urinary FLC/Cr ratio at the lower and upper extremes of the range, as shown in Fig 4. A one SD higher KCR was associated with a HR of 1.58 (1.40–1.79) and a one SD higher LCR was associated with a HR of 1.47 (1.31–1.66). A urinary FLC/Cr ratio above the 75^th^ centile was associated with HRs of 3.18 (2.26–4.47) for KCR and 3.67 (2.62–5.16) for LCR. The cumulative incidence of ESRD by quartiles of urinary FLC/Cr are shown in Fig 5. The univariable associations between other baseline variables and risk of ESRD are shown in Table 3.

**Table 3:** Subhazard ratios with 95% confidence intervals in univariable competing-risk models for risk of end stage renal disease accounting for the competing risk of death.

Variable	Subhazard ratio (95% CI)	*P*
**Age**	0.75 (0.65–0.87)	<0.0001
**Gender**		
Male	0.97 (0.68–1.36)	0.84
Female	1.04 (0.73–1.46)	0.84
**Ethnicity**		
White	0.66 (0.47–0.93)	0.017
South Asian	1.50 (1.03–2.19)	0.036
Black	1.38 (0.82–2.31)	0.23
**Comorbidities**		
Diabetes mellitus	0.90 (0.63–1.30)	0.58
Ischaemic heart disease	0.69 (0.44–1.08)	0.10
Cerebrovascular disease	1.18 (0.69–2.01)	0.55
Peripheral vascular disease	0.73 (0.37–1.43)	0.36
COPD	0.57 (0.29–1.13)	0.11
Malignancy	0.61 (0.34–1.09)	0.10
**Renal diagnosis**		
Ischaemic/hypertensive	0.99 (0.67–1.45)	0.95
Glomerulonephritis	0.85 (0.51–1.41)	0.52
Diabetic kidney disease	1.64 (0.99–2.72)	0.06
Polycystic kidney disease	2.22 (1.34–3.69)	0.002
Interstitial nephropathy	0.71 (0.32–1.59)	0.40
Reflux nephropathy	0.30 (0.05–1.94)	0.21
Other/uncertain	0.90 (0.62–1.32)	0.60
**Estimated GFR**	0.25 (0.17–0.38)	<0.0001
**Urinary ACR**	1.38 (1.20–1.58)	<0.0001
**Systolic blood pressure**	1.28 (1.09–1.49)	0.002
**Diastolic blood pressure**	1.24 (1.04–1.48)	0.015
**Serum κ FLC**	1.67 (1.49–1.88)	<0.0001
**Serum λ FLC**	1.57 (1.40–1.78)	<0.0001
**Serum κ+λ FLC**	1.69 (1.50–1.91)	<0.0001
**Urinary KCR**		
Per +1 SD	1.58 (1.40–1.79)	<0.0001
> 27.7 mg/mmol	3.18 (2.26–4.47)	<0.0001
**Urinary LCR**		
Per +1 SD	1.47 (1.31–1.66)	<0.0001
> 5.1 mg/mmol	3.67 (2.62–5.16)	<0.0001

Urinary KCR and LCR were analysed as both a continuous variable (per +1 SD) and a categorical variable (above versus below the 75^th^ centile). Subhazard ratios for continuous variables represent the risk associated with an increase of one standard deviation.

Abbreviations: ACR = albumin/creatinine ratio; CI = confidence interval; COPD = chronic obstructive pulmonary disease; FLC = free light chains; GFR = glomerular filtration rate; KCR = kappa/creatinine ratio; LCR = lambda/creatinine ratio; SD = standard deviation; SHR = subhazard ratio

Multivariable models incorporating urinary FLC/Cr ratios are shown in Table 4. Urinary KCR and LCR both remained significantly associated with risk of ESRD after adjustment for either eGFR (model 1) or ACR (model 2). However, after adjustment for both eGFR and ACR (model 3), including models incorporating age, sex, ethnicity, renal diagnosis, MAP, eGFR, and ACR (model 4), urinary KCR and LCR as continuous or spline predictor variables were not significantly associated with risk of ESRD. The significant association between urinary FLC/Cr ratios above the 75^th^ centile and risk of ESRD did however remain in models 3 and 4.

**Table 4:** Subhazard ratios with 95% confidence intervals for urinary free light chain/creatinine ratios as continuous and categorical (above 75^th^ centile) predictors in multivariable competing risk models for end-stage renal disease, adjusted for the competing risk of death.

Model	Subhazard Ratio (95% Confidence Interval)			
	Kappa/creatinine ratio		Lambda/creatinine ratio	
	Per +1 SD	> 27.7 mg/mmol	Per +1 SD	> 5.1 mg/mmol
**1**^a^	**1.35 (1.19–1.55)**	**2.22 (1.55–3.18)**	**1.30 (1.14–1.49)**	**2.56 (1.78–3.68)**
**2**^b^	**1.43 (1.23–1.65)**	**2.55 (1.71–3.80)**	**1.32 (1.14–1.54)**	**2.79 (1.85–4.21)**
**3**^c^	1.14 (0.96–1.36)	**1.61 (1.05–2.49)**	0.99 (0.78–1.24)	**1.65 (1.05–2.60)**
**4**^d^	1.22 (1.00–1.50)	**1.74 (1.12–2.71)**	1.00 (0.78–1.27)	**2.05 (1.26–3.33)**

Bold values have a *P* value < 0.05.

^a^Adjusted for eGFR.

^b^Adjusted for urinary ACR.

^c^Adjusted for eGFR and urinary ACR.

^d^Adjusted for age, sex, ethnicity, renal diagnosis, MAP, eGFR, and urinary ACR.

Abbreviations: ACR = albumin/creatinine ratio; eGFR = estimated glomerular filtration rate; FLC = free light chains; MAP = mean arterial pressure; SD = standard deviation.

When these analyses were repeated in participants with a urinary ACR < 30 mg/mmol (*N* = 265), we found that higher urinary FLC/Cr ratios, as either continuous or categorical variables, were associated with a significantly increased risk of ESRD on univariable analysis, but the associations became non-significant after adjustment for eGFR.

### Urinary FLC/Cr in risk stratification

After excluding those who died without ESRD within two years (*N* = 34) and those with less than two years of follow-up (*N* = 58), 464 participants had data on ESRD at two years, of whom 60 (12.9%) had reached ESRD. Measures of model performance for the prediction of ESRD at two years are shown in Table 5, including the incremental value of adding urinary FLC/Cr ratios to KFRE. The baseline model containing only KFRE had a strong predictive ability for ESRD at two years (C-statistic 0.89 [95% CI 0.84–0.94]) and was well calibrated (Hosmer-Lemeshow statistic 12.19, *P* = 0.14). None of the KFRE + KCR or KFRE + LCR models had a significant change in the C-statistic, suggesting no improvement in discrimination between those who did and did not develop ESRD, nor any significant improvement in reclassification of risk based on the IDI. Similarly, the addition of splines of KCR and LCR to the KFRE model did not improve the C-statistic or show any improvement in discrimination based on the IDI.

**Table 5:** Logistic regression models for the prediction of end stage renal disease at two years, with measures of model performance and the incremental value of adding kappa/creatinine ratio (KCR) or lambda/creatinine ratio (LCR) to a baseline model containing the kidney failure risk equation (KFRE).

Statistic	Model				
	Kidney Failure Risk Equation (KFRE)	KFRE + KCR		KFRE + LCR	
		Per +1 SD	> 27.6 mg/mmol	Per +1 SD	> 5.0 mg/mmol
**Odds ratio (95% CI)**					
KFRE	1.10 (1.08–1.13)	1.10 (1.07–1.13)	1.10 (1.07–1.13)	1.10 (1.07–1.13)	1.09 (1.07–1.12)
KCR or LCR		1.05 (0.71–1.55)	1.54 (0.67–3.50)	1.16 (0.75–1.77)	2.67 (1.19–6.03)
***R***^**2**^ **(Nagelkerke)**	0.436	0.437	0.440	0.438	0.457
**Hosmer-Lemeshow (*P*)**	12.19 (0.14)	12.37 (0.14)	9.44 (0.31)	11.71 (0.16)	7.07 (0.53)
**C-statistic (95% CI)**	0.89 (0.84–0.94)	0.89 (0.84–0.94)	0.89 (0.84–0.94)	0.89 (0.83–0.94)	0.90 (0.85–0.95)
**Δ C-statistic**		0.00 (-0.01–0.01)	0.00 (-0.02–0.02)	0.00 (-0.01–0.01)	0.01 (-0.01–0.03)
**IDI**		0.00 (-0.01–0.02)	0.00 (-0.01–0.02)	0.00 (-0.01–0.02)	0.00 (-0.01–0.04)

Models were fitted with LCR and KCR as both continuous predictors (per +1 SD) and with a categorical cut-off above the 75^th^ centile.

Abbreviations: CI = confidence interval; IDI = integrated discrimination improvement; KCR = kappa/creatinine ratio; KFRE = kidney failure risk equation; LCR = lambda/creatinine ratio; SD = standard deviation.

## Discussion

There is significant interest in identifying bioclinical prognostic factors in CKD that might enhance our current models for risk stratification and potentially identify novel therapeutic targets. This is to the best of our knowledge the first study to evaluate whether there is an independent association between urinary FLCs and risk of ESRD in patients with CKD. We utilised a cohort of patients who were prospectively recruited with (i) a high risk of CKD progression; (ii) extended follow-up; (iii) a hard end-point; and (iv) a high proportion progressing to the end-point.

There was a negative correlation between eGFR and urinary FLC/Cr ratios, i.e. FLC/Cr ratios increase as GFR falls, consistent with previously published data [13]. We hypothesise that in patients with CKD, as nephrons are lost, there is hyperfiltration of the remaining functional glomeruli and increased concentrations of FLC in the glomerular filtrate that exceeds the capacity of the proximal tubule to reabsorb and metabolise them. Tubular dysfunction with decreased uptake of FLC may also contribute to increasing urinary FLC/Cr ratios.

There was also a positive correlation between urinary FLC/Cr ratios and ACR. Regardless of the underlying cause of CKD, glomerular damage (associated with albuminuria and possibly with increased filtration of FLCs) often coexists with tubulointerstitial fibrosis (and thus possibly reduced tubular FLC reabsorption). The correlation between urinary albumin and FLC is therefore not unexpected.

Adjustment for the confounding relationships with eGFR and ACR significantly attenuated the association between FLC/Cr ratios as continuous predictor variables and risk of ESRD, which became non-significant. The lack of an independent association may reflect their lack of specificity for renal damage: urinary FLC/Cr ratios correlated most strongly with serum FLC, suggesting that systemic inflammation may be an important determinant of urinary levels, and urinary FLC concentration may also be influenced by mucosal secretion in the urinary tract, although data do not exist on how much this pathway contributes.

Despite the lack of an independent association between FLC/Cr ratios and progression to ESRD when analysed as full-range continuous variables, our multivariable analyses did show that urinary FLC/Cr ratios above the 75^th^ centile were independently associated with a significantly increased risk of ESRD. It is possible that this is due to a nephrotoxic effect of FLCs, i.e. those with the highest levels in the urine may be more likely to sustain FLC-induced tubular damage, thus increasing their risk of CKD progression and ESRD. A nephrotoxic effect of FLCs has been proposed as a possible explanation for an independent association between serum FLC levels and progression to ESRD in patients with CKD, although the published data on the relationship between serum FLC and progression to ESRD are inconsistent [28, 29]. Alternatively, the highest levels may be present in patients who have glomerular hyperfiltration and/or tubular dysfunction, both factors that are associated with accelerated progression of CKD. It is also possible that the association reflects a residual confounding effect of kidney function that has not been fully accounted for by adjustment for creatinine-based eGFR.

Adding urinary FLC/Cr ratios to an established model for the estimation of two-year risk of ESRD did not improve model performance in our cohort of patients with advanced kidney disease. However, having previously been shown to be detectable prior to the development of albuminuria, the use of urinary FLCs to stratify risk in early CKD could be examined in other CKD cohorts with populations made up of less severe CKD.

The strength of this study is that it included a well-characterised cohort of patients with prospective follow-up and a significant number of outcome events. Limitations were that it was an observational study without mechanistic data, and we lacked a separate cohort to validate our findings.

In conclusion, urinary FLCs have an association with progression to ESRD in patients with CKD which appears to be explained largely by their correlation with eGFR and ACR, and the current evidence suggests that measuring urinary FLC does not improve upon current models for risk stratification.
